# A reusable catalyst based on CuO hexapods and a CuO–Ag composite for the highly efficient reduction of nitrophenols†

**DOI:** 10.1039/d1ra01560e

**Published:** 2021-04-08

**Authors:** Nannan Zhang, Yuxi Meng, Yuxue Ning, Andrew E. H. Wheatley, Fang Chai

**Affiliations:** Key Laboratory of Photochemical Biomaterials and Energy Storage Materials, Heilongjiang Province, Key Laboratory for Photonic and Electronic Bandgap Materials, Ministry of Education, College of Chemistry and Chemical Engineering, Harbin Normal University Harbin 150025 Heilongjiang China fangchai@gmail.com; Department of Chemistry, University of Cambridge Lensfield Rd Cambridge CB2 1EW UK

## Abstract

The enormous and urgent need to explore cost-effective catalysts with high efficiency has always been at the forefront of environmental protection and remediation research. This work develops a novel strategy for the fabrication of reusable CuO-based non-noble metal nanomaterials as high-efficiency catalysts. We report a facile and eco-friendly synthesis of CuO hexapods and CuO–Ag composite using uric acid as a reductant and protectant. Both exhibited high catalytic activity in the hydrogenation of 4-nitrophenol (4-NP) to 4-aminophenol (4-AP) by sodium borohydride (NaBH_4_), with the CuO–Ag composite showing superior catalytic performance. Notably, the highest turnover frequency of CuO–Ag reached 7.97 × 10^−2^ s^−1^, which was much higher than numerous noble-metal nanomaterials. In addition, CuO hexapods and CuO–Ag composite were also shown to act as highly efficient and recyclable catalysts in the degeneration of 4-NP. Both CuO hexapods and the CuO–Ag composite exhibited outstanding catalytic durability, with no significant loss of activity over more than 10 cycles in the hydrogenation of 4-NP.

## Abstract 2

Schematic illustration for the process of preparing CuO hexapods and CuO–Ag composite, and their application in catalytically reducing 4-NP and K_3_(Fe(CN)_6_).

## Graphical abstract

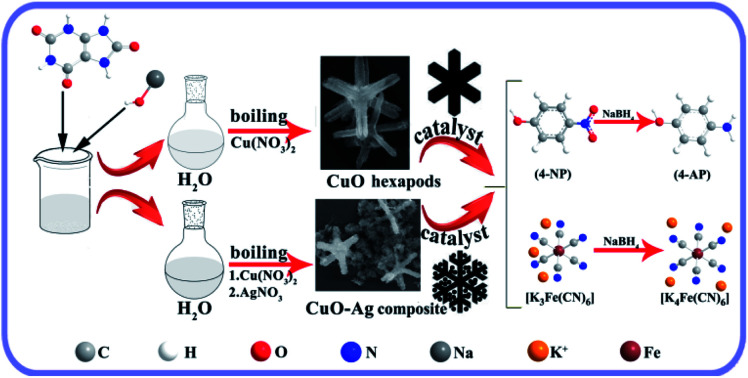

## 1. Introduction

4-Nitrophenol is one of most common nitroaromatic compounds used to produce pesticides, insecticides, herbicides, pharmaceuticals and explosives.^1–3^ However, due to its toxicity and potential carcinogenicity, 4-NP has been identified as a dangerous organic contaminant by the US Environmental Protection Agency.^4^ As such, its detection in and eradication from the environment has become a major target.^5,6^ As a means of eradicating highly toxic 4-NP, its conversion to less toxic derivative 4-AP by catalytic reductive degradation is particularly appealing because of the latter's contribution to the production of various antipyretic and analgesic drugs.^1^

Metallic nanomaterials have been widely investigated in recent years.^7,8^ In particular, numerous works have reported the synthesis of noble metal nanoparticles (NPs), wires, rods and tubes.^9,10^ These have been targeted for their remarkable physical and chemical features and have demonstrated applications in sensing, catalysis, biomedicine, therapy, and optical devices. Noble metal NPs (such as Au, Ag, Pd, *etc.*) have been widely applied as catalysts in the reduction of 4-NP by sodium borohydride,^7,11,12^ which process has been considered valuable because of the potential of the resulting 4-AP as a chemical raw material.^13^ However, the scarcity of reserves of precious metals such as Au, Pt, and Pd, restricts their use in practical fields and makes it essential to explore alternative, more earth-abundant materials. And recently, some non-noble metal (such as Cu, Co, Ni, Zn, *etc.*) based materials have been proved achieving a great effect on hydrogenation of nitroaromatic compounds at ambient temperature.^14,15^

As earth-abundant and inexpensive metal, Cu has been frequently investigated as alternative to rare metal materials, especially in catalysis.^16^ Cu based nanomaterials are particularly attractive due to their improving catalytic activity effectively.^16^ Recently, the development of Cu, CuO, Cu_2_O and Cu-based bimetallic nanomaterials has been reported as a highly appealing means of diluting the use of precious metals by equivalent substitution in the synthesis of nanomaterials for practical catalysis, owing to their low cost, facile preparation method and easy scale-up.^15,17,18^ Multiple ways to explore the preparation of Cu-based nanomaterials that promise to overcome their sensitivity to oxygen, water, and other chemical entities, have been investigated and have lately led to complex structures, such as Cu-based bimetallic NPs, or copper oxides.^19,20^ Wang *et al.* reported Cu NPs supported on activated carbon, which exhibited efficient catalytic activity for selective reduction of vanillin.^21^ Meanwhile, Chen and co-workers reported a Cu/CuO–Ag composite which exhibited excellent activity in the degradation of organic pollutants.^17^ Sui's group explored hexapod Cu_2_O micro-crystals and used them as non-enzymatic sensors for detecting glucose with outstanding results.^22^ And Ma *et al.* prepared bimetallic Cu and Co NP-doped N-containing carbon frameworks, which showed higher catalytic performance than some noble-metal catalysts in reduction of 4-NP.^15^ Cu-based nanomaterials have already demonstrated excellent catalytic efficiency in the hydrogenation of 4-NP, demonstrating cost-effectiveness far superior to that of precious metal catalysts.^23,24^ And it has been reported that Cu can host another noble metal such as Pd, Au, Rh and Ag to extend series of bimetallic nanoparticles. These have exhibited superior catalytic activity in hydrogenation, dehydrogenations, C–C and C–O coupling reactions, *etc.* and this has been attributed to synergistic effects.^25^ Moreover, similar effects where Cu surfaces have partly oxidized have been reported, Cu_2_O/CuO has hosted NPs such as Pd, Ni, and Ag and the resulting composites have displayed remarkable catalytic activity.^26–29^ With its indirect narrow band gap (1.3–1.51 eV),^30^ the CuO has been argued to act as a semiconductor, possessing a strong absorption in the visible region, high carrier concentration and low toxicity. As a visible light-activated photocatalyst, CuO has already exhibited excellent performance in the degradation of organic dyes.^17^ Moreover, its chemical, physical and electronic properties combine with its economic cost, convenient synthesis and stability to make it a good candidate for the reduction of nitroaromatics.^31,32^ CuO as a catalyst points to new avenues in the reduction and degradation of nitrophenols in water.^33^ Of course the efficiency of catalysis could not be compared with that of noble metal NPs such as Au, Ag. Thus further appeal comes from the ability of CuO to support noble metal NPs, offering reduced cost by lowering noble metal content and instead relying on the provision of active metal-support interfaces. In this way, Cu has been reported to enhance the activity of composite catalysts in numerous electron- and photo-catalytic reactions.^16^ For those systems with active noble metal-support interfaces, catalysts with enhanced performance are easily achieved by enlarging the metal-support interfaces.

As a precious metal, Ag has more abundant resources and lower cost than Pt, Pd and Au. Ag can catalyse a range of reductive chemical reactions transforming both organic and inorganic environmental pollutants.^11^ The unique characteristics conductivity and surface area-normalized turnover frequencies of Ag endow it is considered one of the few techno-economically viable alternatives to Pt, such as in oxygen reduction reaction.^34^ However, extending the ideas above on Cu-based heterobimetallic ensembles, density functional theory (DFT) simulations have shown that in the nanoalloy form, CuAg NPs have a high density of states at the Fermi level. These data suggested they might represent compelling alternatives to expensive Pt-based catalysts.^34,35^ This view of the promise of Ag-based nanomaterials in catalysts led,^36^ for instance, Ren and co-workers to report the synthesis of Ag-covered Cu_2_O by galvanic replacement and delivered highly effective CO_2_ reduction.^28^

Here we develop a simple and environmentally friendly strategy for preparing heterocomposites based on the interspersion of CuO hexapods and Ag NPs, as a typical semiconductor load noble NPs, the CuO–Ag composite revealing high catalytic activity. The properties of the as-prepared materials are correlated to the surface properties of the catalyst. We use the hydrogenation of nitrophenol isomers and potassium ferricyanide as model catalytic reactions.^37^ Impressively, the synthesized CuO–Ag composite exhibit excellent catalytic performance, enabling the reduction of 4-NP by borohydride in only 180 s. Excellent reusability of both CuO hexapods and CuO–Ag composite in the reduction of 4-NP has been verified using recycling experiments.

## 2. Experimental

### 2.1 Material

All chemical reagents used are of analytical grade and were obtained from Aladdin Chemical Co., Ltd. (Shanghai). CuO hexapods were synthesized by dissolving 0.0028 g of uric acid using 0.2 mL of NaOH solution (0.1 M) at room temperature. This solution was added to 10 mL of ultrapure water and lightly boiled under magnetic stirring. Then 0.05 mL of 0.1 M copper nitrate solution was added and the mixture lightly boiled for a further 5 min, after which it was left to cool to ambient temperature. After centrifugation (6000 r/min, by using a LG16-B centrifuge), the collected residue was washed three times by ethanol and ultrapure water alternately, and then baked in a vacuum oven at 80 °C for 5 h to yield CuO hexapods. For the synthesis of CuO–Ag composite, the addition of copper nitrate was followed by boiling of the reaction mixture for 5 min, whereupon 0.05 mL of 0.01 M silver nitrate solution (the ratio of Cu : Ag is 10 : 1) in ultrapure water was introduced and light boiling was maintained for 15–20 min. After this time a black precipitate emerged and the mixture was left to cool to room temperature. The CuO–Ag composite was then obtained by centrifugation and washing according to the first procedure.

### 2.2 Instrumentals

X-ray diffraction (XRD) utilized a Rigaku DMax-2600 PC (Japan) diffractometer of 2*θ* range 10–90 by a CuKα source of wavelength, *λ* = 1.5406 Å. Scanning electron microscopy (SEM) images were obtained on Hitachi Su-70, and transmission electron microscope (TEM) images, high-resolution TEM (HRTEM) and energy dispersive X-ray spectroscopy (EDS) were performed by a FEI Tecnai G2 F20 TEM with an accelerator voltage of 200 kV. The TEM sample was prepared by dropping a diluted suspension in ethanol on a Cu grid supported by carbon film. UV-vis spectroscopy was operated on a Shimadzu UV-2600 spectrometer to monitor the reduction of 4-NP. X-ray photoelectron spectroscopy (XPS) was acquired on an AXIS Ultra DLD using monochromatic Al Kα radiation.

### 2.3 Catalytic test

An aqueous solution of 3-NP (or 4-NP) (0.01 M, 0.03 mL) and a NaBH_4_ solution freshly prepared using ice water were mixed in a quartz cuvette with 2.5 mL of ultrapure water, and then 0.03 mL of 1 mg mL^−1^ CuO hexapods (or CuO–Ag composite) dispersion in water was added. The reaction was monitored by UV-vis spectroscopy at 30 s intervals. To estimate the reusability of the catalyst, the reacted solution was removed by pipette, keeping the catalyst in cuvette, and then an equal amount of the reactant solution was supplied for the next cycle, and the above process was repeated. The cycle was repeated 11 times, to guarantee the amount of the catalyst was sufficient during the circulation, 2 mg of catalyst was used in the recycling process.

### 2.4 Hydrogenation of K_3_(Fe(CN)_6_)

1.2 mL ultrapure water was injected into a quartz cuvette. Aqueous solutions of K_3_(Fe(CN)_6_) (0.4 mL, 8 × 10^−3^ M) and NaBH_4_ (1 mL, 0.04 M) were added. Finally, 0.03 mL CuO hexapods (or CuO–Ag composite) suspension in ultrapure water was added.

## 3. Results and discussion

### 3.1 Catalyst preparation and characterization

**Fig. 1:**
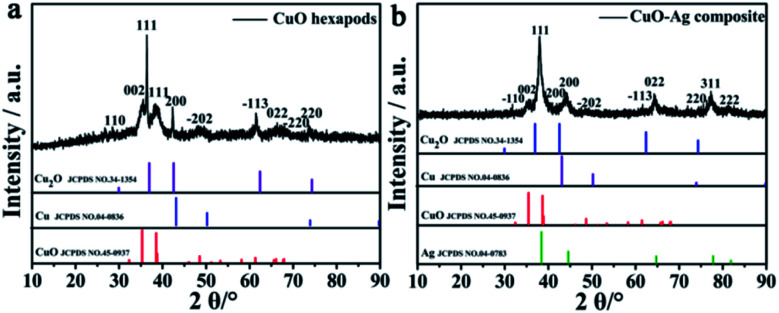
XRD patterns of (a) CuO hexapods and (b) CuO–Ag composite.

**Scheme 1:**
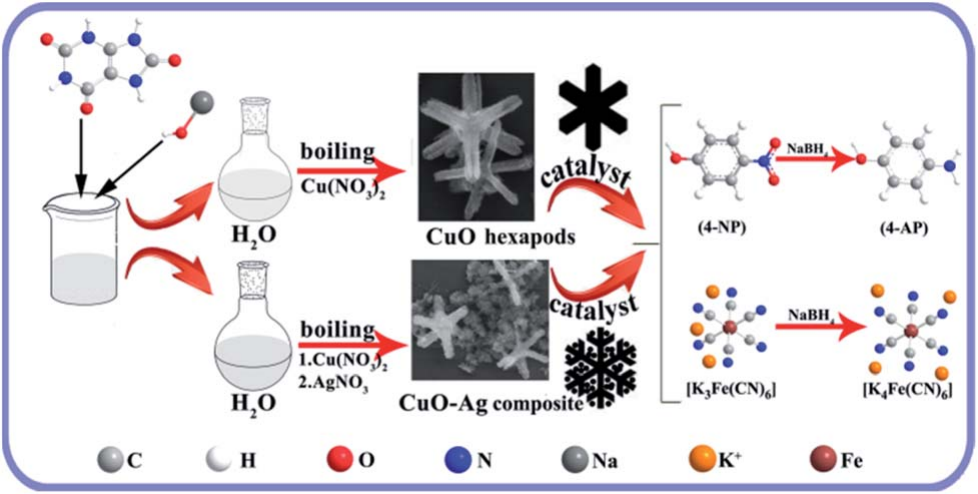
Preparation of CuO hexapods and CuO–Ag composite, and their application in catalytically reducing 4-NP and K_3_(Fe(CN)_6_).

CuO hexapod and CuO–Ag composite catalysts were both synthesized by using a simple one pot method (Scheme 1). For CuO hexapod synthesis, uric acid acted as both reductant and protectant. While the CuO–Ag composite were formed by an analogous method to which AgNO_3_ was added. The component and structure of CuO hexapods were first characterized by XRD. The diffraction pattern (Fig. 1a) exhibited peaks at 35.5°, 38.7°, 48.7°, 53.5°, 65.8° and 68.1°, corresponding to the (002), (111), (−202), (−113), (022) and (−220) crystal planes of Cu(ii) oxide (JCPDS no. 45-0937).^38,39^ Meanwhile, the minor 42.6° and 74.4° signals were assigned to (200) and (311) in a small amount of Cu_2_O (JCPDS no. 34-1354). And there is no obvious diffraction of Cu(0) observed compared with standard PDF card no. 04-0836. The XRD pattern for CuO–Ag composite is displayed in Fig. 1b, the peaks characteristic of CuO now being augmented by peaks diagnostic of elemental Ag. Hence, sharp diffraction peaks at 38.1°, 44.3°, 77.5° and 81.5° were attributable to the (111), (200), (311) and (222) crystal planes of Ag (JCPDS no. 04-0783). Again, small peaks attributable to cuprous oxide (JCPDS no. 34-1354) meant that apart from main CuO, the nanocomposite had incorporated limited Cu_2_O. Overall, XRD analysis pointed to the composition CuO–Ag.

**Fig. 2:**
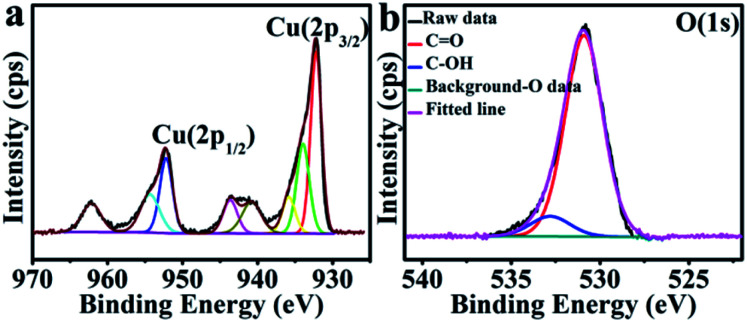
XPS data for CuO hexapods: the high-resolution spectra of (a) Cu 2p and (b) O 1s.

The chemical composition and valence states pertinent to the CuO hexapods and CuO–Ag composite were confirmed by XPS. Fig. S1a† and 2a show the full spectrum and Cu 2p region for CuO hexapods. Deconvolution of the high resolution XP spectrum of the Cu 2p region (Fig. 2a) revealed peaks attributable to Cu 2p_3/2_ and Cu 2p_1/2_ at 932.7 eV and 952.6 eV, pointing to Cu(i) due to the XRD ruled out the existence of Cu(0).^21,40^ Meanwhile, peaks at 934.5, 940.5 and 943.6 eV confirmed the presence of Cu(ii).^17^ These data are consistent with CuO (see XRD discussion). Fig. 2b shows the O 1s XP spectrum, and peaks at 531.2 and 532.7 eV were assigned to C=O and C–OH.^41^ The former was derived from oxygen in the Cu_2_O and CuO phase.^42,43^ Overall, XPS data confirmed the presence of Cu_2_O and CuO, which was compatible with the analysis of the XRD pattern.^44–47^

**Fig. 3:**
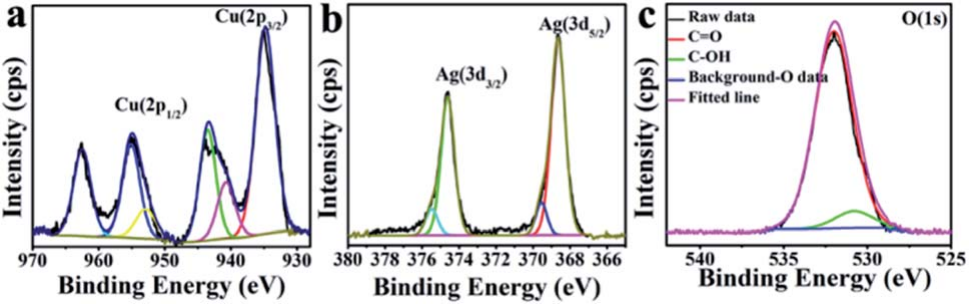
XPS data for CuO–Ag composite: the high resolution spectra of (a) Cu 2p, (b) Ag 3d and (c) O 1s.

The XPS interrogation of CuO–Ag composite revealed five peaks (Fig. S1b†): C 1s, N 1s, O 1s, Ag 3d and Cu 2p at 284.6 eV, 399.3 eV, 532 eV, 368.6 eV and 935 eV. The main Cu 2p peaks were located at 932.8 eV (Cu 2p_3/2_) and 953.2 eV (Cu 2p_1/2_) suggesting, in the same way as for the CuO hexapods, analysis, Cu(i),^21^ (Fig. 3a). These notwithstanding, peaks revealed by deconvolution at 934.5, 940.5 and 943.6 eV were assigned to Cu(ii), indicating the main content to be of CuO.^26,44–47^ Lastly, the high-resolution spectrum of Ag 3d exhibited peaks at 374.4 eV and 368.4 eV (Fig. 3b), which could be attributed to Ag 3d_5/2_ and Ag 3d_3/2_ in Ag(0).^17^Fig. 3c shows the high resolution O 1s spectrum, which on fitting qualitatively established the different bonding states of oxygen: C–OH (531.2 eV), and C=O (532.7 eV).^41^ Overall, the XPS data were compatible with XRD analysis, revealing the presence of Ag and CuO.

**Fig. 4:**
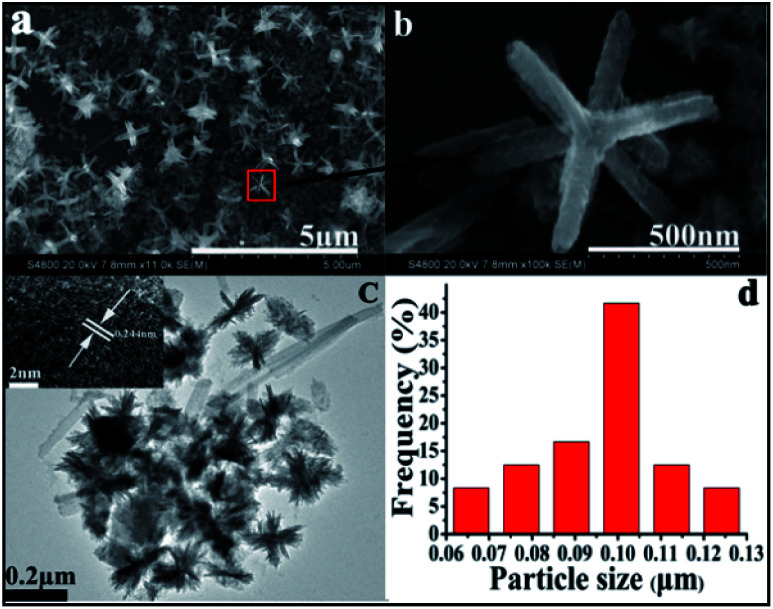
(a) Representative SEM image of CuO hexapods and (b) an enlarged image of the red area in (a). (c) TEM image of CuO hexapods (inset: HRTEM image showing lattice fringes that correspond to (111) for CuO). (d) CuO hexapod size distribution (*N* = 100).

Having investigated composition, SEM and TEM analysis was used to reveal the morphology and structure of both materials. Representative SEM images (Fig. 4a and b) disclose CuO hexapod structures.^22,48^ According to these data, CuO hexapods exhibit rough surfaces and are composed of stacked stick-like nanorods, being attenuated from the particle core to the nanorod tip to yield a distinctive hexapod structure. The assembled CuO hexapods were 424.2 ± 16.7 nm in mean diameter with branches 420 nm, the width is about 85 nm. Both Fig. 4a and b show that alongside these polypods are large numbers of much smaller nanorods with diameters of ∼20 nm and lengths of 80–100 nm, which suggests a growth mechanism for the CuO hexapods. These can be viewed as being composed of tiny CuO nanorods by their constantly pasting on the surface of CuO hexapods based on the ripening mechanism.^49^ The representative TEM image shown in Fig. 4c further illustrates the formation of the CuO hexapods, lots of small needles cluster with the mean diameter of 26.18 ± 0.23 nm were observed, and their half dimeter was about 99.49 ± 1.17 nm (the size distribution histogram of the CuO hexapods is provided in Fig. 4d). Meanwhile some longer pods with length of about 400 nm also can be observed, which was consistent with SEM. HRTEM imaging of CuO hexapods (Fig. 4c, inset) showed lattice fringes with a periodic spacing of 0.244 nm, corresponding to the (111) facet of CuO,^17^ substantiating XRD and XPS data. The EDS (Fig. S2a†) further confirmed the component of CuO of the hexapods.

**Fig. 5:**
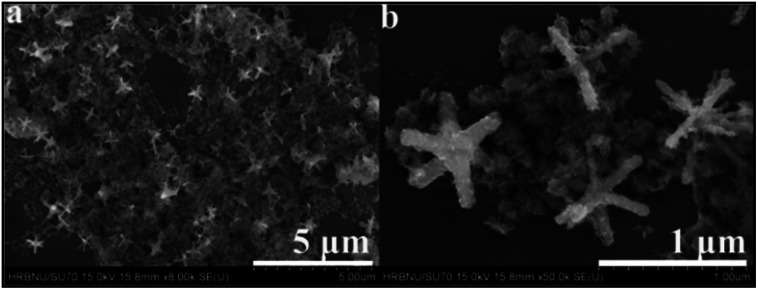
(a) Representative SEM image of CuO–Ag composite and, (b) an enlarged view of CuO–Ag composite.

**Fig. 6:**
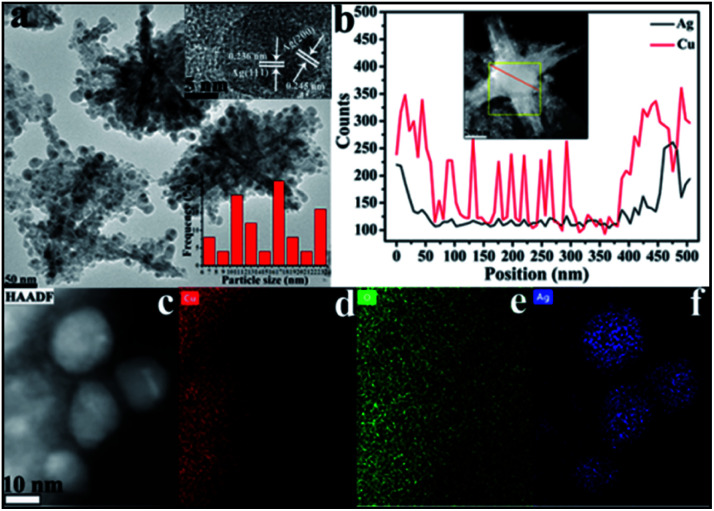
(a) The representative TEM image of the CuO–Ag composite, with little nanoparticle size distribution histogram (bottom right) and HRTEM image showing the lattice spacing between Ag (111) and Ag (200) planes (inset, upper right). (b) EDS line profile analysis of a CuO–Ag composite with (inset) beam location indicated in red and (c) HAADF-STEM image of CuO–Ag composite and corresponding EDS elemental maps of the (d) Cu (red), (e) O (green) and (f) Ag (blue).

Moving to the SEM imaging of CuO–Ag composite (Fig. 5a) the essential CuO hexapod framework has clearly been retained. The rods composing CuO–Ag composite revealed a mean half diameter of 369.6 ± 10.8 nm. A magnified image of CuO–Ag composite (Fig. 5b) shows that the apparent roughness of the polypods surface can be attributed to the dispersal of small nanoparticles over the surface of the hexapod. To illustrate the morphology of composites, the further analysis was carried out by TEM. Fig. S3† and 6a show representative TEM images of CuO–Ag composites. The basic skeleton was akin to that seen for the CuO hexapods, with branches ∼370 nm long. Evidence for Ag^+^ having been reduced to Ag came from analysis of the observation of the surface-coating nanoparticles. These demonstrated a mean particle size of 17.4 ± 0.52 nm. HRTEM imaging and EDS line scans revealed that these had a complex composition and structure. As can be seen in Fig. 6a (inset), three sets of lattice fringes were easily resolvable. The lattice spacings were 0.245 nm and 0.236 nm, which index to Ag (200) and Ag (111),^50^ respectively, confirming the existence of Ag NPs on the suface of hexapods. The distribution of Ag and Cu in CuO–Ag composite was also proved by EDS (Fig. S2b†). Representative high-angle annular dark field and scanning transmission electron microscopy (HAADF-STEM) of a hexapod are shown in Fig. 6b. The inset displays the passage of the beam through the center of an individual composite, with the resulting EDS line profile shown in the main image.

Though both Ag and Cu were detected across the entire skeleton (Fig. 6b), it is clear that the two edges of the snowflake were Ag-rich meaning that Ag resided mostly on the polypod surface. Notably, an individual surface-decorating nanoparticle was also scanned to confirm its composition.^51^ To gain insights into component information of surface nanoparticles, the elemental analysis mapping was further measured. The Fig. 6c–f display the elemental Cu, O and Ag can be observed to distribute different area. Image (d) and (e) shows Cu and O EDX overlays, where Cu shows occupation in areas where O is abundant detected, however, the distribution area of Ag almost does not coincide with that of Cu, confirmed the Ag NPs scattered on the surface of CuO pods.

### 3.2 Preliminary catalytic tests

**Fig. 7:**
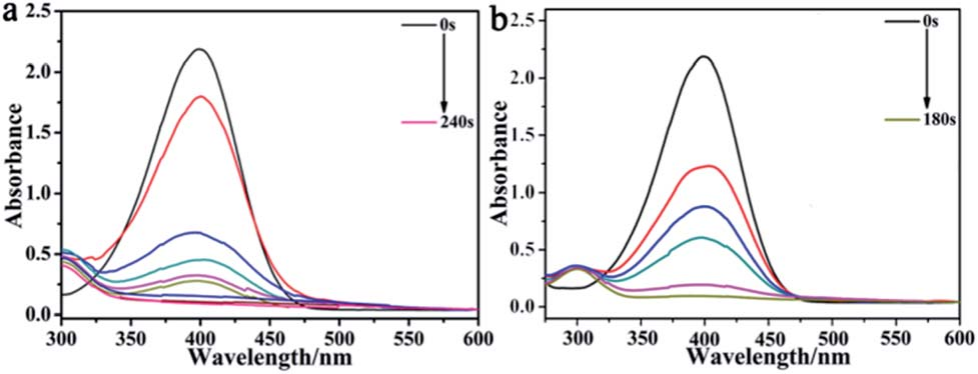
The UV-vis spectra for the reduction of 4-NP by excess NaBH_4_ with (a) CuO hexapods and (b) CuO–Ag composite as catalyst.

The process of catalysis is described in the ESI,† where the failure of 4-NP reduction by NaBH_4_ illustrated the importance of the catalyst (Fig. S4 and S5†). In contrast, the CuO hexapods catalysed reduction of 4-NP by excess NaBH_4_, reaching completion in 240 seconds (Fig. 7a), during which time the sample lost its yellow colouration. A pseudo-kinetic first equation was applied, whereby ln(*C*_*t*_/*C*_0_) = ln(*A*_*t*_/*A*_0_) = −*k*_app_*t* (*A*_*t*_ = absorption at time *t*, and *A*_0_ = absorption without catalyst), where *k*_app_ is the apparent rate constant, and *C*_0_ and *C*_*t*_ correspond to the concentration of 4-NP initially and at time *t*, respectively. The linear plot of ln(*C*_*t*_/*C*_0_) as *t* (Fig. S6a,† inset) followed a first-order kinetic model (*R*^2^ = 0.9651),^52,53^ giving *k*_app_ = 1.3 × 10^−2^ s^−1^, which established the CuO hexapods has highly catalytic efficient in this reaction.^4,54^

The corresponding performance CuO–Ag composite, which showed the same core hexapod structure as the CuO catalysts, is shown in Fig. 7b. The same reduction of 4-NP was now accomplished in 180 s, with *k*_app_ = 1.7 × 10^−2^ s^−1^ (Fig. S6b†), indicating the superior activity of CuO–Ag composite. These data suggested that Ag-doping the CuO hexapods improved the electrical properties of the catalyst. Having established that Ag provided an enhancement in activity, the effect of doping was probed to more fully understand the reasons for improved performance in the presence of Ag. To do this, the performance of composites incorporating different ratios of Ag/Cu was investigated.^55–58^

### 3.3 Ag-doping studies

To probe the morphological and structural effects of varying Ag levels, the Ag : Cu molar ratio in the heterobimetallic composite synthesis was varied from 0.1–1.0. SEM analysis (Fig. S7†) showed that the morphology of the resulting material evolved with the change of content. As reported above, when the molar ratio of Ag : Cu was 0.1, the nanocomposites co-existed with many Ag NPs (Fig. S7a†). When the amount of Ag increased to Ag : Cu = 0.5 (Fig. S7b†), the background of Ag nanoparticles became more significant and incorporated a small number of much larger nanoparticles. And it is difficult to notice pod structure in Fig. S7c,† on account of the overwhelming presence of numerous Ag NPs. The EDS was carried out to compare their component in the nano composites (Fig. S8†). Due to the Ag NPs concentrated on the surface of the composited materials, the amount of Ag in three ratio materials collected by EDS is 19.60%, 42.18% and 47.12% (Table S1†) respectively, reflected the different content of Ag NPs. The effect of catalysts with varying the relative amounts of the two metals in the reduction of 4-NP was next investigated (Fig. S9†). Compared with the Ag : Cu = 0.1 sample, the 0.5 and 1.0 catalysts exhibited better catalytic activity, completing the process of reduction in 150 and 120 s, respectively, and allowing *k*_app_ to be calculated as 1.9 × 10^−2^ s^−1^ and 2.7 × 10^−2^ s^−1^. These data are much higher than many reported results.^4,18^ To further deliberate on the catalytic activity of CuO hexapods and CuO–Ag composite (including different ratio), the relative catalyst data have been compared. It can be noticed from Table S2† that the catalytic efficiency of these two catalysts perform comparably with or better than previously reported noble metal catalysts, with turnover frequencies (TOFs) of 4.03 × 10^−2^ s^−1^ and 7.97 × 10^−2^ s^−1^ (Ag : Cu = 0.1) respectively. The excellent catalytic activity of the latter is consistent with the inclusion of heterojunctions and the synergistic electric effect they enable, with Cu exhibiting a higher electron chemical potential relative to Ag, whereas Ag has higher electron conductivity than Cu.^59–61^ These data are in line with prior research, which has indicated that a small variation in the local electronic structure at the interface between Ag and Cu can enhance catalytic activity.^59,60^ Comparing catalysts with varying Ag : Cu molar ratios (Table S2†) revealed only small increments in performance, Ag : Cu = 0.5 (TOF = 8.23 × 10^−2^ s^−1^) and 1.0 (TOF = 8.55 × 10^−2^ s^−1^), indicated a small amount of Ag doping can achieve high cost performance.

**Fig. 8:**
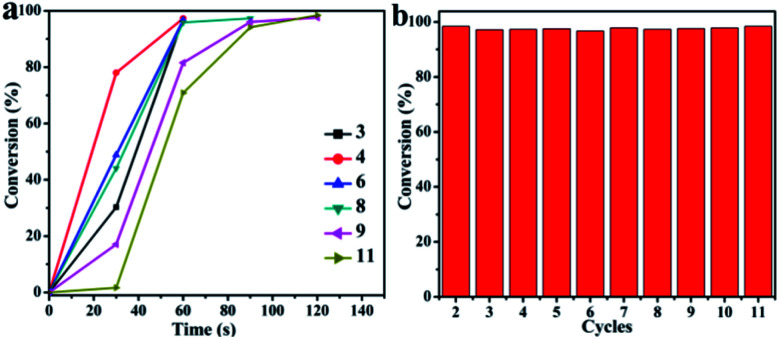
(a) Conversion (%) of 4-NP as reaction time over CuO hexapods for multiple catalytic cycles. (b) Conversion (%) for the reduction of 4-NP with NaBH_4_ over CuO hexapods for each cycle.

Reusability is a key factor in the development of new catalysts for applications. Therefore, the recycling ability of each of the catalysts developed here was tested. The conversion of 4-NP using CuO hexapods was in excess of 95% in repeated 11 cycles as shown in Fig. 8. Recycling experiments used 0.002 g catalyst. Whereas the first cycle reached completion essentially instantly, cycles 2–4 did so in less than 60 s (Fig. S10a–c†). Clearly, with successive cycles, the time taken to complete the reduction reaction gradually rose and, after more than 10 turns, 4-NP was only fully catalytically reduced by CuO hexapods after 180 s. As shown in Fig. S12a and b,† though the pods significantly reduced compared with the as-prepared CuO hexapods, some pods structure still can be surveyed after more than 10 cycles catalysis, exhibited their good stability. Though other pods changed to smaller particles, the composition did not change which can be confirmed by EDS (Fig. S12b†). The stable conversion efficiency for hydrogenation of 4-NP over 10 cycles (Fig. 8) points to CuO hexapods being a reliable and easily recycled catalyst for 4-NP reduction under mild conditions.

**Fig. 9:**
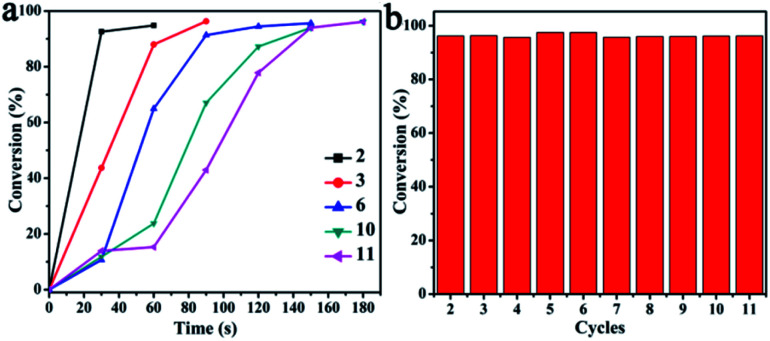
(a) Conversion (%) of 4-NP as reaction time over CuO–Ag composite for multiple catalytic cycles. (b) Conversion (%) of 4-NP with NaBH_4_ over CuO–Ag composite for each cycle.

The recyclability of the CuO–Ag composite catalyst was investigated using a similar procedure to that employed for CuO. The catalytic reduction of 4-NP with CuO–Ag composite was successfully accomplished more than 10 cycles as shown in Fig. S11.† The CuO–Ag composite was used continuously for 11 cycles with, once again, the reduction completing after increasing, albeit brief, reaction durations. After 11 cycles, reaction reached completion in 180 s. As shown in Fig. 9, over 95% conversions were achieved in all recycling experiments. The recovered catalysts were also characterized by the SEM and EDS (Fig. S12c and d†), which indicated the similar morphology and main component of CuO–Ag composite remained after more than 10 turns, demonstrating an excellent reusable catalyst for 4-NP reduction.

To further investigate and compare their catalytic activities, CuO hexapods and CuO–Ag composite were applied to the reduction of 3-NP and K_3_[Fe(CN)_6_]. Fig. S13a† depicts the spectroscopic data obtained using 0.03 mL of CuO hexapods suspension (1 mg mL^−1^) as catalyst. The reduction reaction of 3-NP was completed within 120 s and from these data ln(*A*_*t*_/*A*_0_) as reaction time reveals good linearity. The kinetic rate constant was calculated to be 1.9 × 10^−2^ s^−1^ (Fig. S14a†). In comparison, under the same conditions CuO–Ag composite completed the reduction of 3-NP within 150 s (Fig. S13b†) with a rate constant of 1.4 × 10^−2^ s^−1^ (Fig. S14b†), which failed to match the performance of CuO hexapods.^4^

To extend the remit of this work beyond the treatment of organic pollutants, we also used NaBH_4_ to reduce K_3_(Fe(CN)_6_) in an inorganic model reaction. In the absence of either catalyst, it took more than 24 h for the reduction reaction to proceed completely. After adding 0.03 mL CuO hexapod suspension (1 mg mL^−1^) as catalyst, the reaction completed quickly, with the absorption at 420 nm eliminated within 180 s (Fig. S15a†), these data producing a rate constant of 1.0 × 10^−2^ s^−1^ (Fig. S16a,† inset). As shown in Fig. S15b,† when an equivalent amount of CuO–Ag composite was used as catalyst, the reaction proceeded more rapidly, giving a rate constant of 1.8 × 10^−2^ s^−1^ (Fig. S16b†). Results show that the catalyst CuO–Ag composite exhibits superior activity in this reaction.

Overall, the catalytic activities of the two materials reported here show strong similarities (Table S3†). In particular, CuO hexapods and CuO–Ag composite were both highly active with 4-NP, yielding almost equivalent first-order rate constants of 1.3 × 10^−2^ s ^−1^ and 1.7 × 10 ^−2^ s ^−1^. The slightly superior activity of CuO–Ag composite was tentatively attributable to synergy between copper and silver. However, catalytic performances differed more significantly for 3-NP and K_3_(Fe(CN)_6_). In the first case CuO hexapods were more effective, whilst in the second case CuO–Ag composite dominated. Generally though, the experimental results showed that the action of the prepared CuO hexapods largely mirrored that of CuO–Ag composite, which is exciting for practical applications.

## 4. Conclusions

In summary, a facile one-step and green protocol for CuO hexapods and CuO–Ag composite synthesis is reported, using low cost uric acid as a reducing agent. Analysis revealed that CuO formed hexapod-shaped frameworks and that Ag nanoparticles ornamented the CuO skeleton in CuO–Ag. Both CuO hexapods and CuO–Ag composite exhibited significant catalytic effects in the reduction by NaBH_4_ of 3-NP, 4-NP and K_3_(Fe(CN)_6_) under mild conditions. The reaction of 4-NP could be accomplished within 120–240 s. For either catalyst, simple recycling more than 10 times led to no obvious loss of catalytic activity, indicating good reusability for each system. Notably, the use of CuO hexapods and CuO–Ag composite catalysts offers scope in reducing dependence on precious metals, and encourages the exploitation of novel architectures that demonstrate cost effectiveness, high catalytic activity and superior reusability.

## Conflicts of interest

There are no conflicts to declare.

## Supplementary Material

RA-011-D1RA01560E-s001
