# Supplementary material for: A reusable catalyst based on CuO hexapods and a CuO–Ag composite for the highly efficient reduction of nitrophenols
Source: RSC Adv. 2021 Apr 8;11(22):13193–200. doi: 10.1039/d1ra01560e (PMC8697534; doi:10.1039/d1ra01560e)
Supplement: RA-011-D1RA01560E-s001 [file RA-011-D1RA01560E-s001.pdf]

Electronic Supplementary Material (ESI) for RSC Advances.
This journal is © The Royal Society of Chemistry 2021

Electronic Supporting Information

A reusable catalyst based on CuO and CuO-Ag composite for the highly efficient reduction of nitrophenols

Nannan Zhang [a], Yuxi Meng [a], Yuxue Ning [a], Andrew E. H. Wheatley [b], Fang Chai [*ab]

a Key Laboratory of Photochemical Biomaterials and Energy Storage Materials, Heilongjiang Province; Key Laboratory for Photonic and Electronic Bandgap Materials, Ministry of Education, College of Chemistry and Chemical Engineering, Harbin Normal University, Harbin, 150025, Heilongjiang, China. E-mail: fangchai@gmail.com

b Department of Chemistry, University of Cambridge, Lensfield Rd, Cambridge CB2 1EW, England.

As shown in Fig. S1a, the survey spectrum clearly displays four peaks at 284.6 eV, 398.7 eV, 530.8 eV and 932.15 eV, attributed to C 1s, N 1s, O 1s and Cu 2p respectively, and these data affirmed the existence of Cu (52.1%), O (25.1%), N (6.9%), and C (15.2%) in the CuO hexapods. And the XPS interrogation of CuO-Ag composite revealed five peaks (Fig. S1b): C 1s, N 1s, O 1s, Ag 3d and Cu 2p at 284.6 eV, 399.3 eV, 532 eV, 368.6 eV and 935 eV.

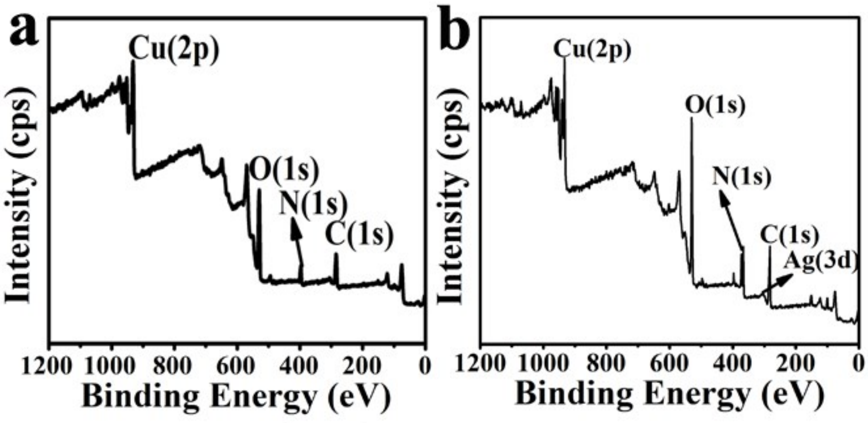

Fig. S1. XPS data for (a) CuO hexapods survey spectrum and (b) CuO-Ag composite survey spectrum.

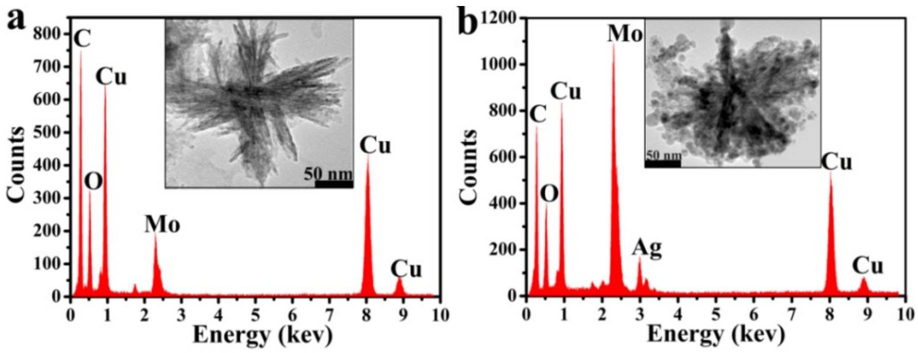

Fig. S2. The EDS analysis of (a) CuO hexapods and (b) CuO-Ag composite. Inset: corresponding representative TEM images, (Mo attribute to the sample support).

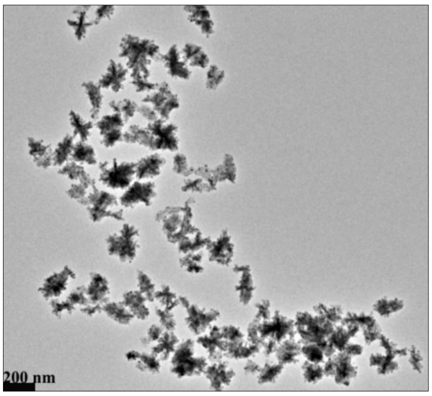

Fig. S3. TEM image of the CuO-Ag composite (large scale).

Hydrogenation of 4-NP in the presence of $NaBH_4$ was carried out at room temperature to evaluate the catalytic activity of CuO hexapods and CuO-Ag composite. Aqueous 4-NP displays a UV-vis absorption maximum at 317 nm, which shifts to 400 nm upon adding freshly prepared aqueous $NaBH_4$ at room temperature. This is accompanied by a color transformation from light yellow to dark yellow and can be attributed to the formation of 4-nitrophenolate ions.[1] However, there is no sign of the reaction happening even with strong reducing agent $NaBH_4$ within 12 hours (Fig. S4). In contrast, the addition of 0.03 mL of CuO hexapods (1 mg/mL) induces a much more rapid conversion. The appearance of an absorption at 300 nm is assigned to 4-AP.[2] To assess the catalytic efficiency the predetermined calibration curve of 4-NP has been confirmed in Fig. S5 firstly.

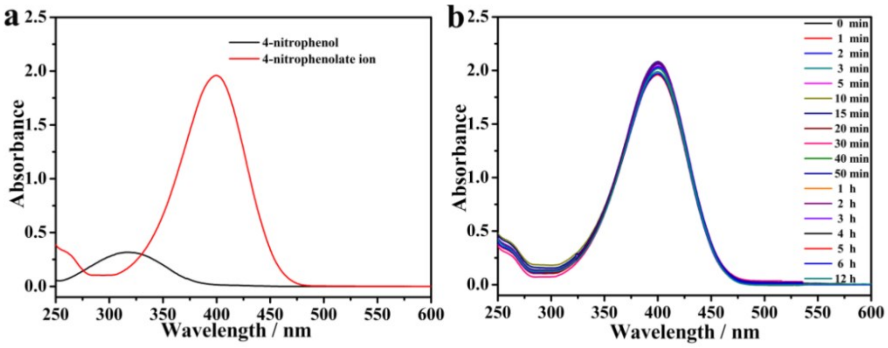

Fig. S4. The UV-vis peaks characteristic of (a) 4-NP before and after addition excessive $NaBH_4$ solution, time-dependent UV-vis absorption spectrum of 4-NP in the presence of $NaBH_4$ solution (b) without catalyst.

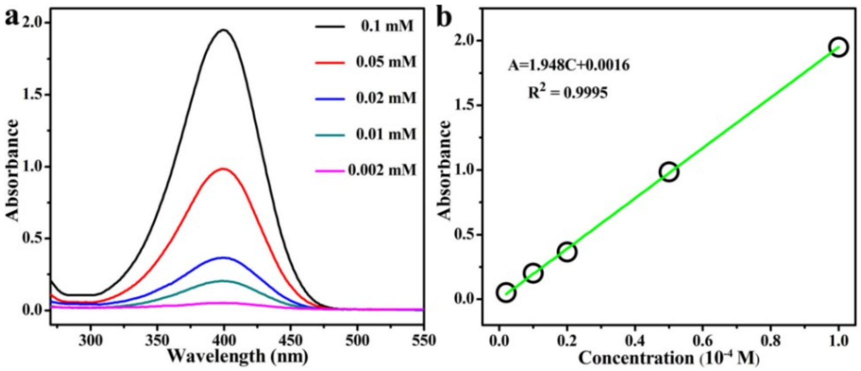

Fig. S5. Absorption spectra of aqueous mixture solutions of 4-NP and $NaBH_4$ at different concentrations of 4-NP, (B) Plot of the peak absorbance against the concentration of 4-NP.

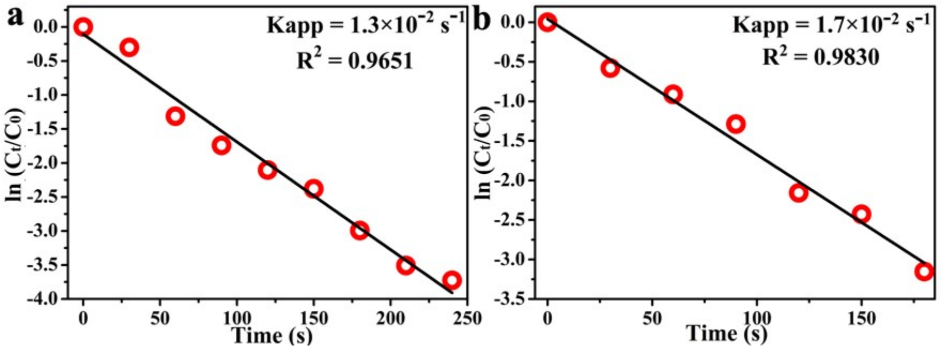

Fig. S6. The plots of ln (C_t/C_0) against time for 4-NP (a) CuO hexapods and (b) CuO-Ag composite as catalyst.

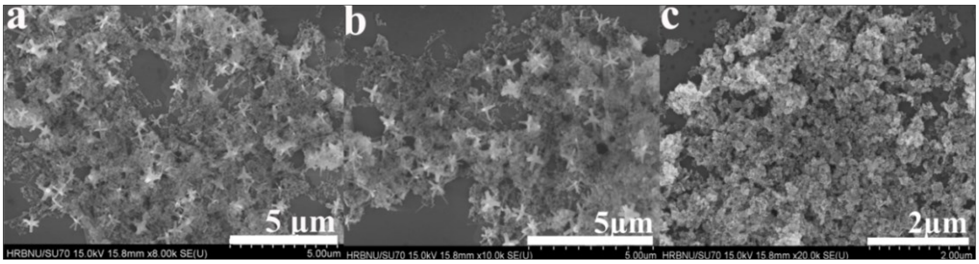

Fig. S7. SEM images of the CuO-Ag composite with different molar ratios (n_{Ag}/n_{Cu}) of (a) 0.1, (b) 0.5, (c) 1.

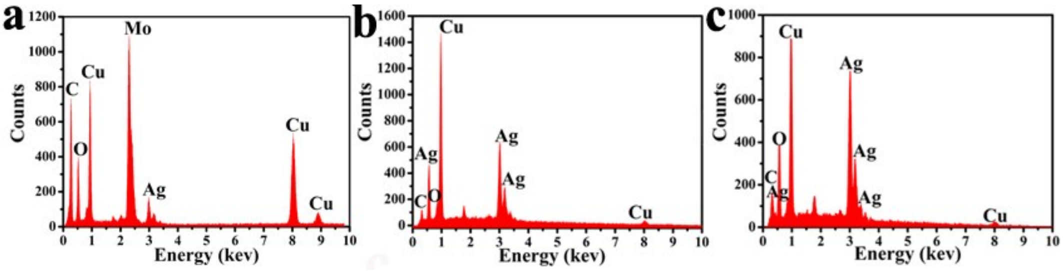

Fig. S8. The EDS analysis using CuO-Ag composite as catalyst with different molar ratios (n_{Ag}/n_{Cu}) of (a) 0.1, (b) 0.5, (c) 1.

Table S1 The percentage of analyte (Cu or Ag) in different samples.

Sample	Analyte	Concentration (mg/L)	At.%
CuO hexapods	Cu	8.027	41.16
CuO-Ag composite	Cu	7.535	38.64
	Ag	3.828	19.63

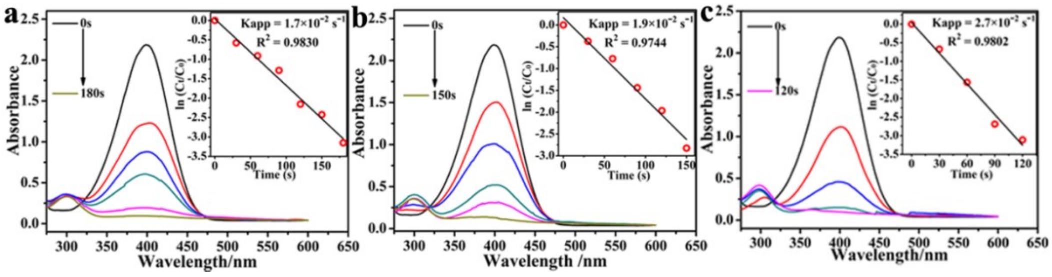

Fig. S9. The UV-vis spectra for the reduction of 4-NP using CuO-Ag composite as catalyst with different molar ratios (n_{Ag}/n_{Cu}) of (a) 0.1, (b) 0.5, (c) 1. Insets: plots of $\ln(C_t/C_0)$ against time for either process.

Table S2 The comparison of kinetic rate constant k and turnover frequency (TOF) value of catalytic $NaBH_4$ reduction of 4-NP by various catalysts.

Catalyst	Amount of catalyst (mg/mL)	Concentration of 4-NP (mol/L)	Reaction time (s)	k value (s^{-1})	TOF[a] (s^{-1})	Reference
UA-Ag NPs	-	1.1×10^{-4}	210	0.0772	-	[3]
Co@NCF	10	1.0×10^{-4}	180	0.0171	1.25×10^{-2}	[4]
highly branched Pt@Ag NPs	1	1.0×10^{-4}	480	0.0059	-	[5]
PdNiP/RGO	1	5.0×10^{-3}	180	0.0235	-	[6]
hematite/Au MS	2	1.0×10^{-4}	390	0.0112	-	[7]
Pd_5Au_5/NCB	1	7.2×10^{-4}	600	0.0048	-	[8]
PCP@Au-Ag composites	1	1.0×10^{-4}	720	0.0028	3.60×10^{-2}	[9]
CeO_2@Au@CeO_2 - MnO_2	5	3.0×10^{-7}	1380	0.0032	2.55×10^{-2}	[10]
Fe_3O_4@Au-Pd NPs	2.8	1.0×10^{-4}	160	0.0289	8.78×10^{-2}	[11]
Fe_3O_4@Pd NPs	2.8	1.0×10^{-4}	280	0.0145	3.28×10^{-2}	[11]
CuO hexapods	1	1.1×10^{-4}	240	0.0130	4.03×10^{-2}	This work
CuO-Ag composite (Ag:Cu = 0.1)	1	1.1×10^{-4}	180	0.0171	7.97×10^{-2}	This work
CuO-Ag composite (Ag:Cu = 0.5)	1	1.1×10^{-4}	150	0.0190	8.23×10^{-2}	This work
CuO-Ag composite (Ag:Cu = 1.0)	1	1.1×10^{-4}	120	0.0271	8.55×10^{-2}	This work

[a]TOF is defined as the ratio of the amount of 4-NP converted substance to the product of the amount of metal substance and time.

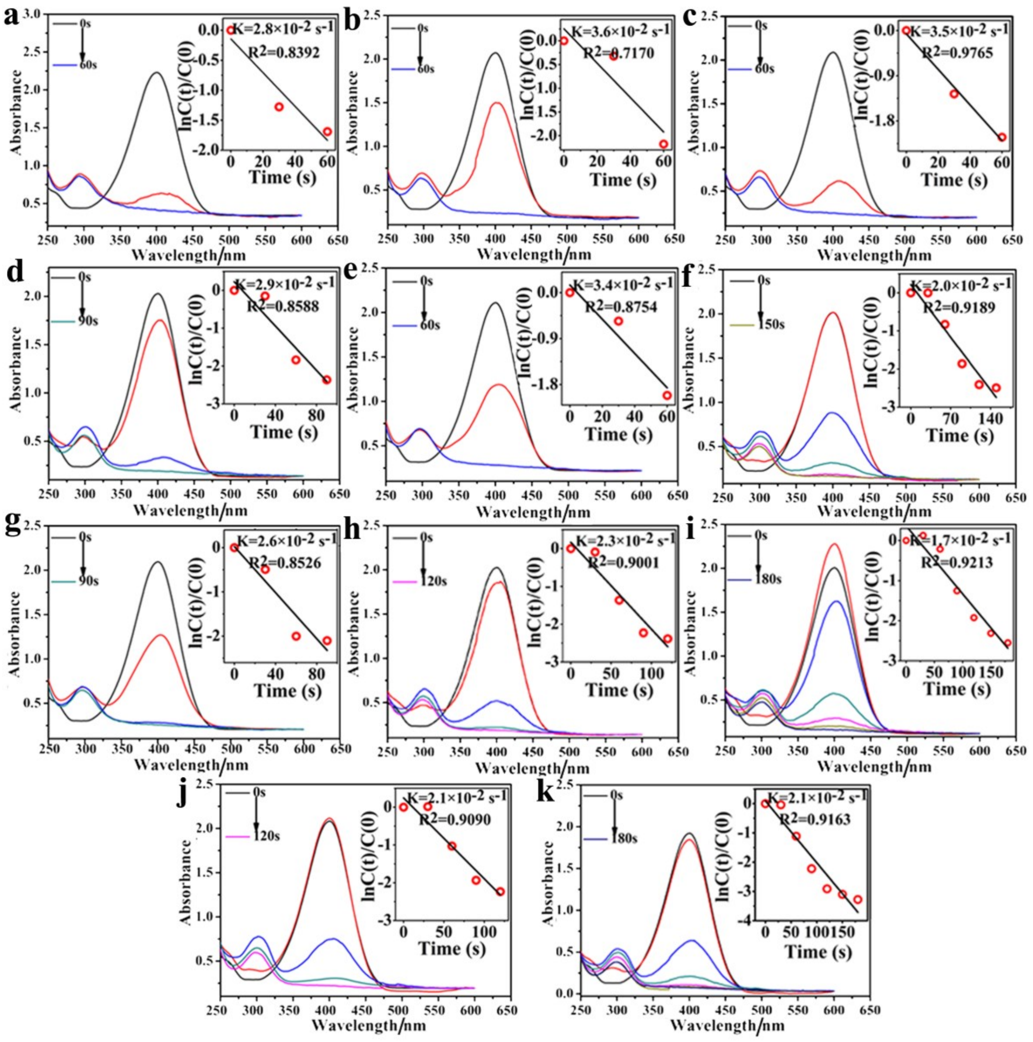

Fig. S10. UV-vis absorption spectra monitoring the reduction of 4-NP by $NaBH_4$ with CuO hexapods recorded for the 2nd (a), 3rd (b), 4th (c), 5th (d), 6th (e), 7th (f), 8th (g), 9th (h), 10th (i), and 11th cycles (j). Inset: $\ln(C_t/C_0)$ as reaction time.

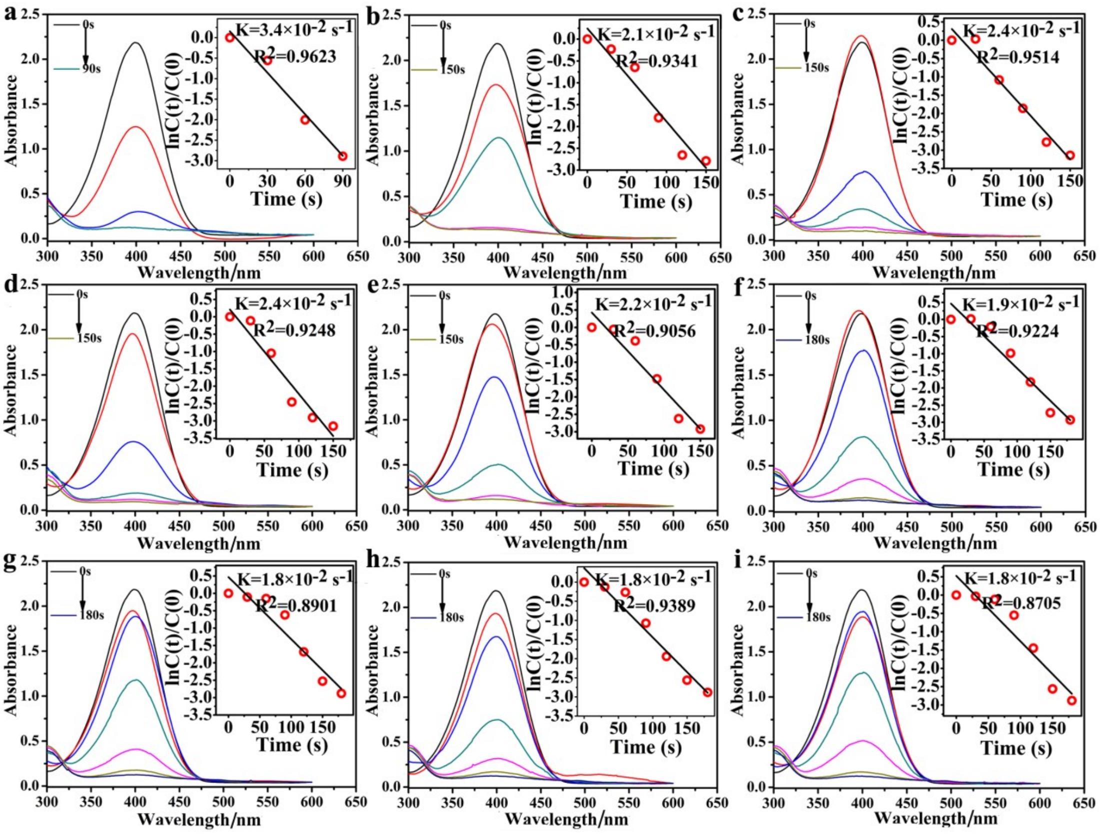

Fig. S11. UV-vis spectra for the reduction of 4-NP by $NaBH_4$ with catalyst CuO-Ag composite recycled for the 3rd (a), 4th (b), 5th (c), 6th (d), 7th (e), 8th (f), 9th (g), 10th (h), 11th (i) time. Insets: the corresponding ln (C_t/C_0) versus reaction time plots.

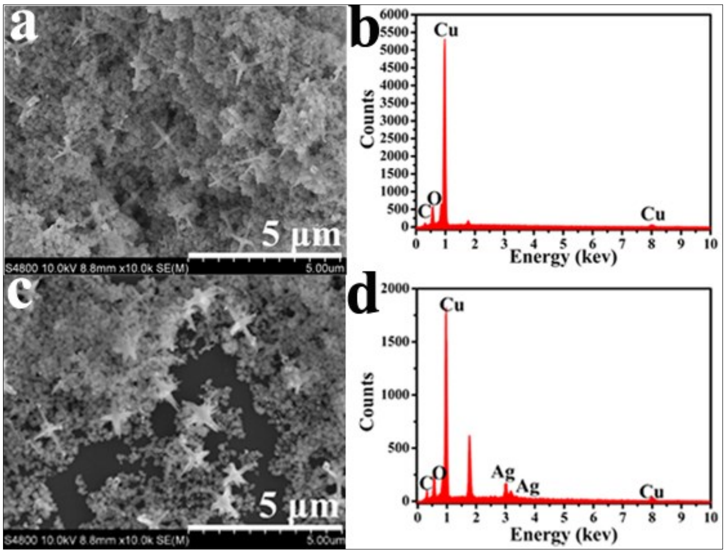

Fig. S12. SEM image (a) and EDS analysis (b) of recovered CuO hexapods after cycling for 12 turns, and SEM image (c) and EDS analysis (d) of recovered CuO-Ag composite after 11 rounds. The corresponding EDS analysis of recovered CuO hexapods and (d) CuO-Ag composite.

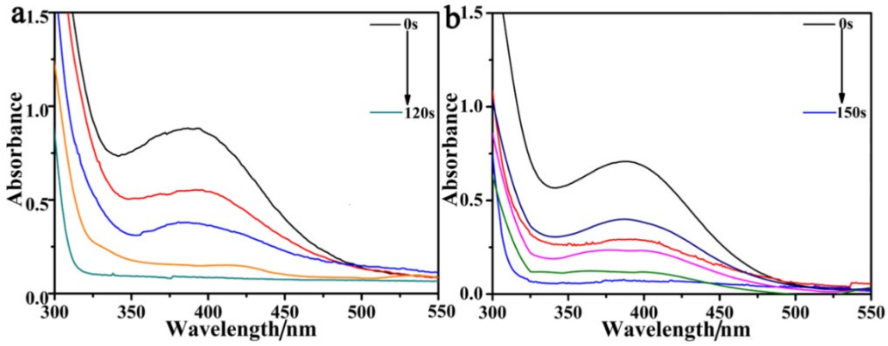

Fig. S13. UV-vis spectra for the reduction of 3-NP by $NaBH_4$ with the catalyst of (a) CuO hexapods and (b) CuO-Ag composite.

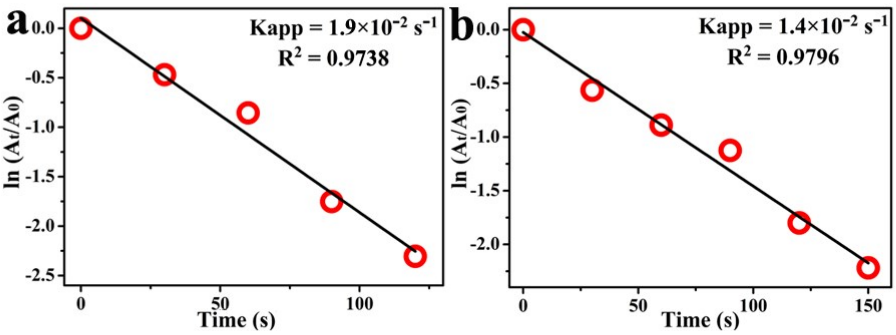

Fig. S14. The corresponding plots $\ln(A_t/A_0)$ versus reaction time for reduction of 3-NP (a) CuO hexapods and (b) CuO-Ag composite as catalyst.

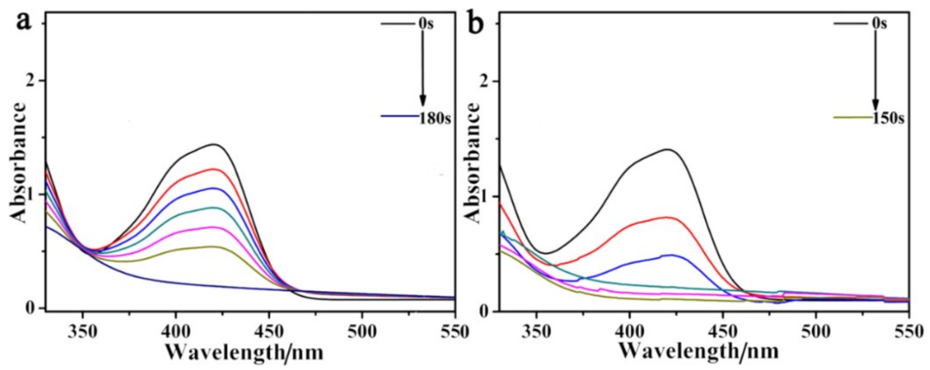

Fig. S15. UV-vis absorption spectra for the reduction of $K_3[Fe(CN)_6]$ by $NaBH_4$ in the presence of (a) CuO hexapods and (b) CuO-Ag composite.

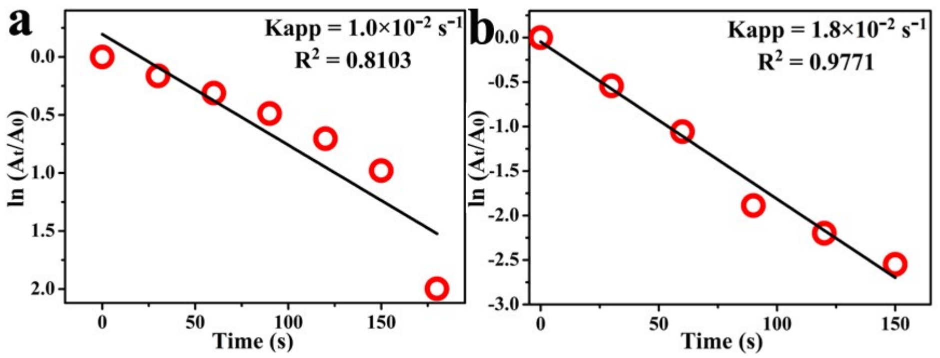

Fig. S16. The corresponding plots $\ln(A_t/A_0)$ versus reaction time for reduction of $K_3Fe(CN)_6$ (a) CuO hexapods and (b) CuO-Ag composite as catalyst.

Table S3. The first order rate constants of several catalysts catalyzed $NaBH_4$ to reduce the 4-NP, 3-NP and $K_3Fe(CN)_6$.

Materials	CuO hexapods	CuO-Ag composite	Ag NPs[a][3]
4-NP	1.30×10^{-2} s^{-1}	1.71×10^{-2} s^{-1}	1.76×10^{-2} s^{-1}
3-NP	1.96×10^{-2} s^{-1}	1.43×10^{-2} s^{-1}	2.56×10^{-2} s^{-1}
$K_3Fe(CN)_6$	1.01×10^{-2} s^{-1}	1.80×10^{-2} s^{-1}	0.95×10^{-2} s^{-1}

a The Ag NPs was synthesized by same method by using uric acid as a reductant and protectant.

References

[1] F. Y. Li, J. H. Feng, Z. Q. Gao, L. Shi, D. Wu, B. Du, Q. Wei, *ACS Appl. Mater. Inter.*, 2019, **11**, 8945-8953.

[2] N. Pradhan, A. Pal, T. Pal, *Colloids Surf. A: Physicochem. Eng. Asp.,* 2002, **196,** 247-257.

[3] Q. J. Dai, T. Wei, C. L. Lv, F. Chai, *Anal. Methods.,* 2018, **10**, 4518-4524.

[4] C. S. Chu, S. Rao, Z. F. Ma, H. L. Han, *Appl. Catal. B-Environ.,* 2019, **256**, 117992.

[5] Z. Lv, X. Zhu, H. Meng, J. Feng, A. Wang, *J. Colloid Interf. Sci.,* 2019, **538**, 349-356.

[6] X. Gao, H. Zhao, Y. Liu, Z. Ren, C. Lin, J. Tao, Y. Zhai, *Mater. Chem. Phys.,* 2019, **222**,391-397.

[7] S. Manivannan, S. An, J. Jeong, M. Viji, K. Kim, *ACS. Appl. Mater. Inter.,* 2020, **12**, 17557-17570.

[8] F. Y. Han, J. W. Xia, X. L. Zhang, Y. S. Fu, *Rsc. Adv.,* 2019, **9**, 17812-17823.

[9] J. W. Fu, S. M. Wang, J. H. Zhu, K. Wang, M. Gao, X. Z. Wang, Q. Xu, *Mater. Chem. Phys.,* 2018, **207**, 315-324.

[10] G. Chen, Y. Wang, Y. Wei, W. Zhao, D. Gao, H. Yang, C. Li, *ACS Appl. Mater. Inter.,* 2018, **10**, 11595-11603.

[11] Q. D. Xia, S. S. Fu, G. J. Ren, F. Chai, J, J. Jiang, F. Y. Qu, *RSC Adv.,* 2016, **6**, 55248.
